# Differences in DNA Methylation and Functional Expression in Lactase Persistent and Non-persistent Individuals

**DOI:** 10.1038/s41598-018-23957-4

**Published:** 2018-04-04

**Authors:** Milena N. Leseva, Richard J. Grand, Hagen Klett, Melanie Boerries, Hauke Busch, Alexandra M. Binder, Karin B. Michels

**Affiliations:** 1 Institute for Prevention and Cancer Epidemiology, Faculty of Medicine and Medical Center, University of Freiburg, Freiburg, Germany; 2 Division of Gastroenterology and Nutrition, Boston Children’s Hospital, Harvard Medical School, Boston, Massachusetts, USA; 3 Institute of Molecular Medicine and Cell Research, University of Freiburg, Freiburg, Germany; 4 German Cancer Consortium (DKTK), Freiburg, Germany; 5 German Cancer Research Center (DKFZ), Heidelberg, Germany; 6 Obstetrics and Gynecology Epidemiology Center, Department of Obstetrics, Gynecology and Reproductive Biology, Brigham and Women’s Hospital, Harvard Medical School, Boston, Massachusetts, USA; 7 Department of Epidemiology, Harvard School of Public Health, Boston, Massachusetts, USA; 8 Luebeck Institute of Experimental Dermatology - Institute for Cardiogenetics, Luebeck, Germany

## Abstract

In humans the expression of lactase changes during post-natal development, leading to phenotypes known as lactase persistence and non-persistence. Polymorphisms within the lactase gene (*LCT*) enhancer, in particular the −13910C > T, but also others, are linked to these phenotypes. We were interested in identifying dynamic mediators of *LCT* regulation, beyond the genotype at −13910C > T. To this end, we investigated two levels of lactase regulation in human intestinal samples obtained from New England children and adolescents of mixed European ancestry: differential expression of transcriptional regulators of *LCT*, and variations in DNA methylation, and their relation to phenotype. Variations in expression of CDX2, POU2F1, GATA4, GATA6, and HNF1α did not correlate with phenotype. However, an epigenome-wide approach using the Illumina Infinium HM450 bead chip identified a differentially methylated position in the *LCT* promoter where methylation levels are associated with the genotype at −13910C > T, the persistence/non-persistence phenotype and lactase enzymatic activity. DNA methylation levels at this promoter site and CpGs in the *LCT* enhancer are associated with genotype. Indeed, taken together they have a higher power to predict lactase phenotypes than the genotype alone.

## Introduction

Lactase non-persistence, also known as adult-type hypolactasia, is the molecular basis of the inability to hydrolyze the lactose found in milk^1,2^. In most mammals, physiological down-regulation of the lactase gene (*LCT*) occurs around the time of weaning and results in low lactase activity throughout adult life^2,3^. The molecular mechanisms of this early life switch from lactose tolerant-to-intolerant phenotype are not completely understood. While lactase does not persist in the majority of the human population, certain ethnic groups have undergone an evolutionary adaptation allowing them to digest lactose even in adulthood^4,5^. However, the molecular underpinnings of this sustained lactase gene expression continue to be only partially explained.

On one hand, genetic variations may play a regulatory role. In Northern Europeans, for example, the T-allele of the −13910C > T intronic variant (rs4988235) located 13,910 bps upstream of the *LCT* transcription start site has been associated with the trait of lactase persistence^6,7^. On the other hand, although it is generally accepted that there is a strong association between the ancestral -13910*C allele with lactase non-persistence and the variant -13910*T allele with lactase persistence in people of European descent, in some cases these genotypes are not completely predictive of the phenotype^8^. Yet, the physiological change in lactase gene expression occurs in the context of a stable DNA sequence. This suggests the presence of dynamic mediators of *LCT* regulation such as epigenetic modifications and/or transcriptional changes.

The small intestine absorbs molecules from the intestinal lumen through enterocytes, the predominant cell type of the intestinal columnar epithelium that possess microvilli to increase surface area for digestion. Microvillous enzymes hydrolyze oligo- and disaccharides to monosaccharides^9^, among them lactose, the disaccharide found in milk, which is hydrolyzed by the apical membrane-anchored glycoprotein known as lactase phlorizin hydrolase (LPH). The different cell types of the intestine are replenished through continuous differentiation of intestinal stem cells (ISCs) in the crypt. The extent of DNA methylation dynamics during this process in humans and during postnatal intestinal maturation is largely unknown, because of inaccessibility of tissue over different time points. It is only recently that the role of DNA methylation changes during the development from fetal to pediatric intestinal epithelium, and their association with disease, has begun to be understood^10^. In the context of mouse intestinal development, Dnmt1-mediated maintenance of methylation has been shown to be essential for crypt ISC differentiation^11–13^. Indeed, genes such as mouse lactase (*Lct*) are induced upon differentiation to enterocytes, with a corresponding decrease in DNA methylation at specific CpG sites^11^. Dnmt1 is also required during mouse intestinal maturation postnatally^14^. Because of the high degree of conservation in expression patterns and regulatory mechanisms, it was feasible that DNA methylation also plays an important role in regulation of human *LCT*. This was confirmed by Labrie *et al*. who identified epigenetically controlled regulatory elements where differential DNA methylation accounted for inter-individual differences of lactase mRNA level in a Lithuanian cohort of individuals^15^. In that study, microarray-based interrogation of DNA modifications across human chromosome 2 and subsequent targeted bisulfite padlock-probe sequencing of the genomic region encompassing *LCT* were used to identify a total of 35 CpGs, clustered in 7 regions where enterocyte-specific DNA methylation differences could be detected. Two of these, namely *MCM6* intron 13 (where rs4988235 resides) and *MCM6* exon 16, were shown to be differentially methylated in CC vs. C/T vs. TT individuals. While the authors were able to demonstrate genotype-dependent changes in DNA methylation and an association between methylation variation and lactase mRNA levels, their analysis did not take into account LPH enzymatic activity levels in designation of the lactase persistence/non-persistence phenotypes. To our knowledge, although lactase enzyme activity and *LCT* mRNA expression levels are highly correlated, there is no known established cut-point for lactase gene expression in determination of persistence vs. non-persistence.

Recently, Baffour-Awuah *et al*. have investigated the association between the genotype at rs4988235 and phenotypic markers such as *LCT* mRNA expression and LPH enzymatic activity in a heterogeneous cohort of children and adolescents mostly of European ancestry^8^. Here, we used a subset of samples from this cohort to identify additional levels of lactase regulation contributing to the switch from lactase persistence to non-persistence that occurs early on in life. We have chosen a method for analysis of DNA methylation that is commonly used in epigenome-wide association studies and provides the opportunity for discovery of differentially methylated positions^16,17^. Through regression modeling we show that DNA methylation in the enhancer and promoter site of the *LCT* gene, rather than differential regulation of intestinal transcription factors, e.g. CDX2, POU2F1, GATA4/6 or HNF1α, is predictive of lactase persistence/non-persistence. Although not the first investigation of DNA methylation as a molecular mechanism for the regulation of *LCT* expression^15^, we have performed the first genome-wide DNA methylation profiling using intestinal tissues obtained from children and adolescents, with the youngest being 8 years old.

## Results

### RT-qPCR analysis of intestinal transcription factors reveals no differential expression in lactase persistent vs. non-persistent individuals

Based on their importance for *LCT* gene regulation, we investigated differential expression of the transcription factors (TFs) GATA4, GATA6, HNF1α and CDX2 between lactase persistent and non-persistent individuals. We further included POU2F1 (also known as OCT1) as it binds preferentially to the rs4988235 T-allele, thereby stimulating expression from the *LCT* promoter^18^. We quantified the TF expression from n = 79 tissue biopsies using RT-qPCR, wherein we defined lactase persistence and non-persistence based on lactase enzymatic activity being greater or less than 15 U/g protein, respectively^1,8^. Among lactase persistent individuals, the median enzymatic activity was approximately 32 U/g protein, ranging from the cut-off of 15 U/g to 73.7 U/g protein (Supplementary Figure S1a).

**Figure 1:**
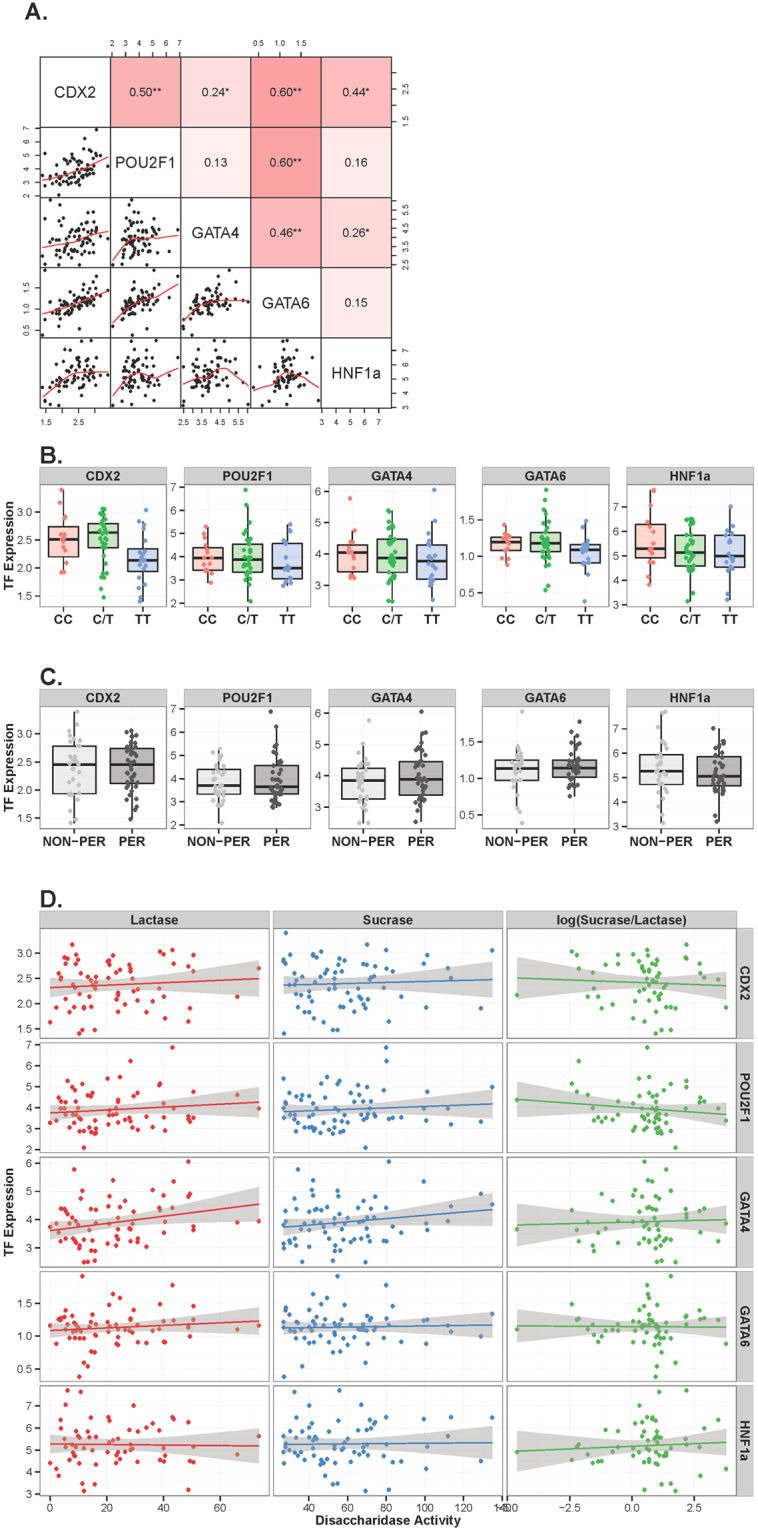
RT-qPCR analysis for major intestinal transcription factors (TF) in lactase persistent and non-persistent individuals. (**A**) Spearman correlation coefficients (upper triangle) and scatter plots (lower triangle) between relative expression levels of CDX2, GATA4/6, POU2F1 and HNF1α. The intensity of color corresponds to the relative strength of the associations (*p < 0.05; **p < 0.001). (**B)** Relative expression of transcription factors CDX2, GATA4/6, POU2F1 and HNF1α in individuals belonging to CC, C/T or TT genotypes at rs4988235 normalized to beta-ACTIN and GAPDH. (**C)** Relative expression of transcription factors CDX2, GATA4/6, POU2F1 and HNF1α in lactase persistent and non-persistent individuals. (**D)** Association between TF expression and disaccharidase enzymatic activity (enzymatic activity data from Baffour-Awuah *et al*.^8^).

We observed moderate to strong positive Spearman correlations among transcription factor mRNA levels ranging from ρ = 0.44 to 0.60 (p < 0.05; Fig. 1A), which is not surprising given that the above TFs are essential for gut development and intestinal stem cell differentiation^19–24^. The strongest correlations were observed between CDX2, POU2F1, and GATA6. Weaker, yet significant associations were observed between GATA4 and CDX2, as well as between GATA4 and HNF1α expression levels (ρ = 0.24 and ρ = 0.26, respectively). Interestingly, CDX2 expression levels were associated with genotype, being significantly lower in TT homozygous individuals (p = 0.022; Fig. 1B). None of the other TFs were associated with ethnicity, gender, age, genotype, lactase mRNA expression or lactase persistence/non-persistence status (Fig. 1C). However, increased lactase enzymatic activity was significantly associated with higher levels of GATA4 expression (p = 0.01; Fig. 1D), after adjusting for gender and age. Although speculative, one explanation for this result might be that GATA4 could potentially act as a positive regulator of genes important for LPH processing and activation, however, the identity of such putative GATA4-targeted genes currently remains elusive. As expected, *LCT* gene expression in the healthy human intestine is unlikely to depend on the above TFs alone, confirming that the level of transcription factor expression cannot explain inter-individual variation in *LCT* expression. Neither is lactase persistence nor non-persistence associated with TF expression levels, which must therefore depend on additional regulators.

### Genome-wide DNA methylation analysis reveals differentially methylated positions (DMPs)

The genotype at −13910C > T (CC or TT) is a strong predictor of the lactase persistence/non-persistence phenotype (p = 0.0001, Chi-squared test). However, its predictive power for the prevalent mixed C/T genotype in this cohort is weak (p = 0.5). Thus, we decided to investigate the epigenetic contribution to *LCT* regulation and potential association with adult-type hypolactasia as an additional regulatory mechanism beyond the genome and intestinal TF expression levels.

**Figure 2:**
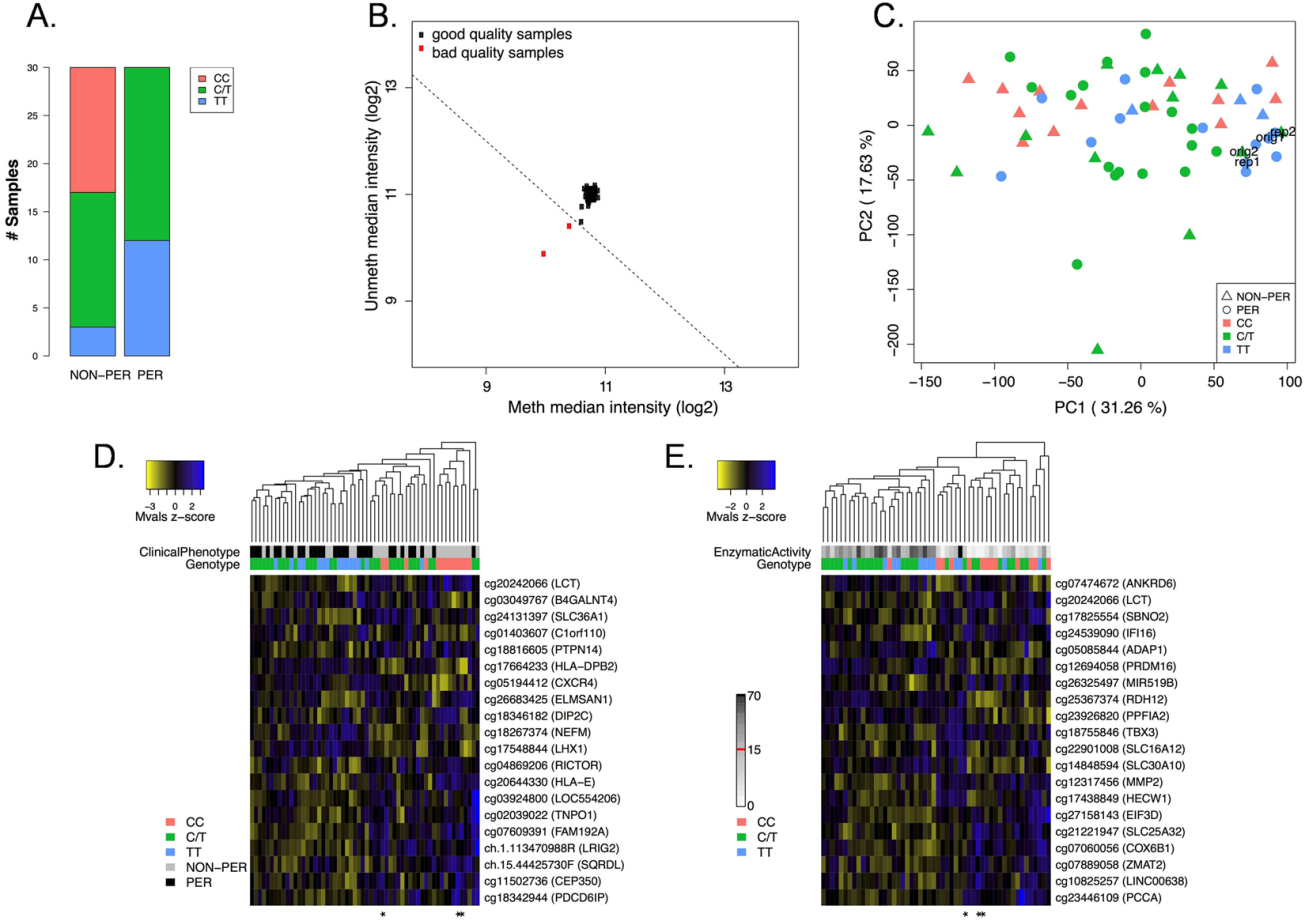
Genome-wide DNA methylation analysis of lactase persistent and non-persistent individuals. (**A**) Composition of samples included on the 450K DNA methylation array. The number of samples in each group, non-persistent (NON-PER) and persistent (PER), is given according to their genotype at −13910C > T (rs4988235). (**B)** Outlier prediction following background correction, dye-bias normalization and filtering: all probes with a detection value p > 0.01 in at least 10% of the data; probes with bead count ≤ 3; and probes with known SNPs. (**C**) PCA plot of DNA methylation variation at all CpGs, excluding CpGs on sex chromosomes (X and Y) after batch correction using ComBat. (**D**–**E)** Colorspace corresponds to methylation levels as z-score transformed M-values (yellow = hypo-, blue = hypermethylation). (**D)** Top 20 DMPs identified following ordinal regression of genotype (CC = 0, C/T = 1, TT = 2); (**E)** Top 20 DMPs identified from ordinal regression analysis using quartiles of enzymatic activity levels (q1 = [0, 6.8], q2 = [6.8, 14.8], q3 = [14.8, 28.6], q4 = [28.6, 73.7]). Both analyses were adjusted for sex, age and estimated surrogate variables. Nearest gene of the DMPs is displayed in parenthesis. A *designates the three self-reported lactose-intolerant individuals (all CC genotype).

While epigenetic mechanisms facilitate phenotype without a corresponding change in DNA sequence, their contribution to *LCT* regulation in the human intestine has been explored by only one study to date, which focused on a 100 kb region surrounding the *LCT* and *MCM6* genes^15^; accordingly, we analyzed genome-wide DNA methylation in a subset of patients (n = 60), half of which were phenotypically lactase persistent, and half non-persistent according to the clinical definition described previously^1,8^. The patients belonged to the three possible genotypes at rs4988235: homozygous CC (n = 13), homozygous TT (n = 15), and heterozygous C/T (n = 32). The sample characteristics are summarized in Fig. 2A. Interestingly, only 3 patients (all CC genotype) had self-reported lactose intolerance. An explanation for this is that chronic ingestion of milk will induce lactose fermenting colonic microflora, thereby ameliorating symptoms of gastrointestinal discomfort. To investigate whether or not the lactase persistent phenotype or rs4988235 genotype are associated with epigenetic differences, we performed a genome-wide DNA methylation analysis using the Illumina Infinium HM450 bead chip. We excluded 6 patients due to low sucrase activity ≤20U/g from all further phenotype analysis^25^. To control for technical variation within the chip analysis we performed two patient sample replicates. Additional characteristics of patients included in the DNA methylation analysis are summarized in Table 1. It should be emphasized that most of the individuals in this heterogeneous cohort are of European ancestry, however, there are some with African, Asian or mixed descent. Detailed ancestry information for each subject can be found in Supplementary Table 1.

**Table 1:** Characteristics of study population used in DNA methylation analysis by Infinium 450K methylation microarray and Pyrosequencing including outlier samples.

Characteristic	Lactase Persistent (N = 30)	Lactase Non-persistent (N = 30)
Average age	15.1 yrs	14.03 yrs
Ethnicity		
* European ancestry*	n = 24	n = 23
* Other*	n = 6	n = 7
Gender		
* Female*	n = 13	n = 17
* Male*	n = 17	n = 13
Genotype −13910C > T (rs4988235)		
* CC*	n = 0	n = 13
* C/T*	n = 18	n = 14
* TT*	n = 12	n = 3

We are not aware of any clinical feature that might be responsible for the lactase non-persistence phenotype of the three TT individuals (see Table 1). It should be noted that these non-persistent TT individuals have an average sucrase enzymatic activity level that is lower than the average for the persistent TT individuals (33.7 U/g and 56.3 U/g, respectively). However, the average S/L ratio was 2.9 for the non-persistent TT’s and 1.69 for the persistent TT’s, respectively. In addition, there was a recent study by Goodrich *et al*. which suggested an association between gut *Bifidobacterium* content and the lactase gene locus, and might also help explain the discrepancy between phenotype and genotype observed for these non-persistent TT individuals^26^.

Following background correction and data normalization, two samples were identified as outliers and removed from further analysis based on the median methylated and unmethylated intensities (Fig. 2B, labeled in red). Although all samples entered the Infinium data production pipeline as a single batch and were run in parallel, principal component analysis (PCA; excluding CpGs on the X- and Y-chromosomes) did reveal batch effects across the lanes of the Illumina 450K chip that can be seen from the deviation of technical replicates (Supplementary Figure S2). Therefore M-values were adjusted using ComBat^27^. While PCA analysis on corrected M-values did not reveal any phenotypic or genotypic clustering, batch effects were efficiently removed (Fig. 2C).

We used Surrogate Variable Analysis (SVA) to control for any unmodeled sources of DNA methylation heterogeneity. When estimating the number of surrogate variables, we identified 12 corresponding to the number of confounding factors. Adjusting for these factors and modeling the possible genotypes at −13910C > T as an ordinal covariate according to the number of T-alleles (i.e. CC = 0, C/T = 1, TT = 2) revealed cg20242066 among the 20 top-ranked DMPs (ranked 10^th^; Fig. 2D). Furthermore, cg20242066 was among the 20 top-ranked DMPs (ranked 5^th^) when testing the association with LPH activity using quartiles of enzymatic activity levels (q1 = [0, 6.8], q2 = [6.8, 14.8], q3 = [14.8, 28.6], q4 = [28.6, 73.7]) and controlling for 12 sources of potential bias, as suggested by the SVA (Fig. 2E). In order to account for inter-sample differences in sucrase enzymatic activity levels, we also adjusted for sucrase in the linear model. This did not dramatically change the top DMP hits identified from the 450K methylation array with cg20242066 now ranked 13^th^ for the genotype analysis (Supplementary Figure S3a) and 3^rd^ for phenotype (LPH enzymatic activity; Supplementary Figure S3b).

**Figure 3:**
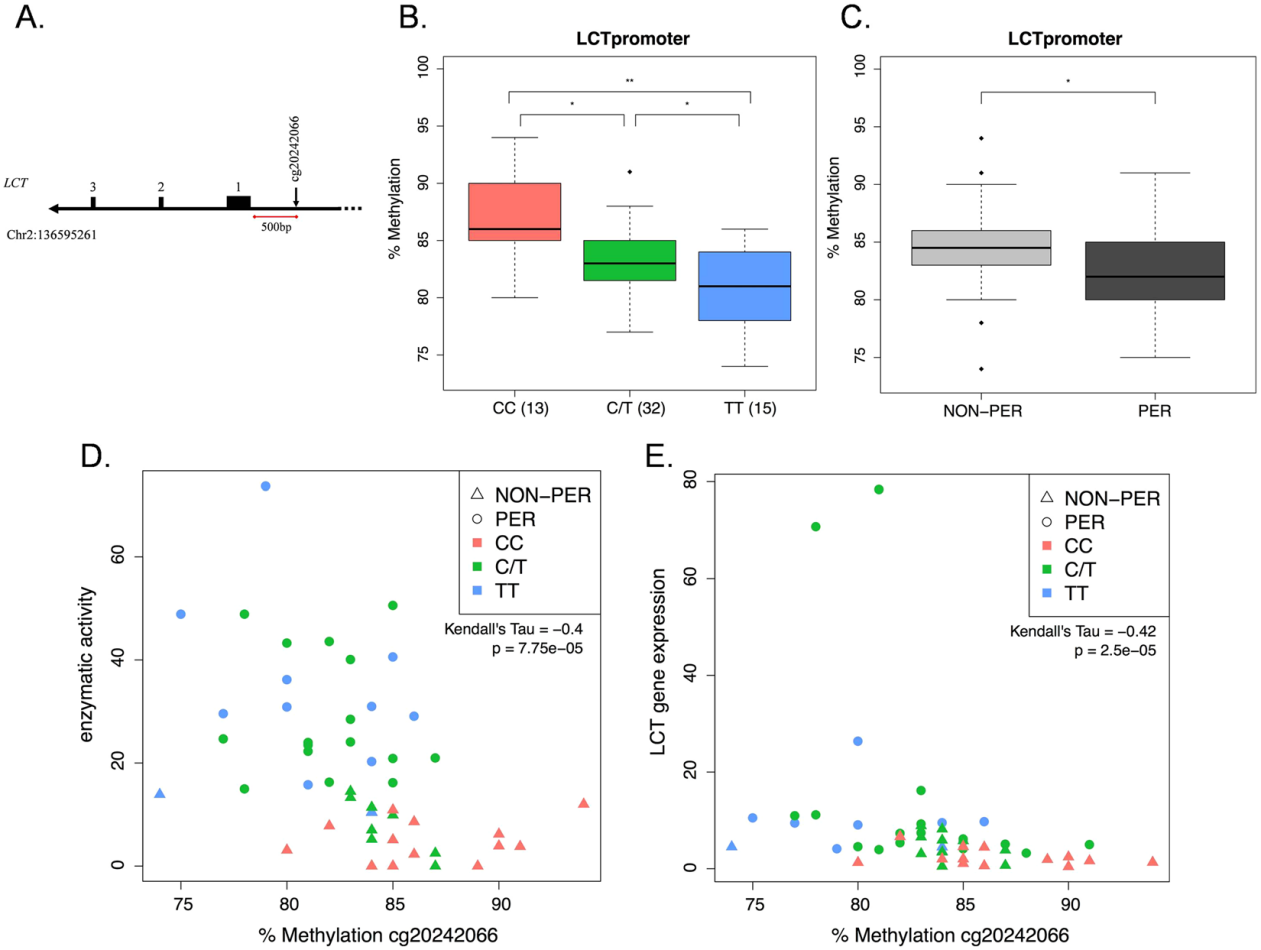
Pyrosequencing of DNA methylation at cg20242066. (**A**) Schematic of *LCT* proximal promoter with location of cg20242066 upstream of the first three exons of the gene. The genomic coordinate of cg20242066 is also given (genome build hg19). (**B)** DNA methylation quantification in CC vs. C/T vs. TT individuals, as well as in (**C)** Phenotypically Persistent (PER) and Non-persistent (NON-PER) individuals. Student’s T-test was calculated for inter-group comparisons (*p < 0.05; **p < 0.001). Number of individuals in each group is given in parenthesis. (**D)** Correlation between DNA methylation at cg20242066 and lactase enzymatic activity (enzymatic activity data from Baffour-Awuah *et al*.^8^). (**E)** Correlation between DNA methylation at cg20242066 and *LCT* gene expression (*LCT* gene expression data from Baffour-Awuah *et al*.^8^).

Cg20242066 is located approximately 500 bps upstream of *LCT* within the proximal promoter region (Fig. 3A). We additionally verified the results from the Illumina bead chip by pyrosequencing at cg20242066. In agreement with the methylation array, sequencing found the cg20242066 site to be differentially methylated in CC vs. C/T vs. TT individuals and lactase persistent vs. non-persistent individuals (Fig. 3B,C). Kendall’s Tau correlation coefficients suggested a negative correlation of the methylation at cg20242066 with both the genotype (correlation = −0.40, p = 0.00018) and the phenotype according to clinical definition (correlation = −0.36; p = 0.0016). We further detected significant negative correlations between methylation and the lactase enzymatic activity (correlation = −0.40 p = 7.75E-05; Fig. 3D), and between methylation and *LCT* gene expression (correlation = −0.42, p = 2.5E-05; Fig. 3E). The pyrosequencing assay covered an additional CpG next to cg20242066 (see Supplementary Table 2), however, it did not appear to be differentially methylated in CC vs. C/T vs. TT individuals, or in persistent vs. non-persistent individuals. Taken together, these results suggest a strong association of methylation at cg20242066 with the −13910C > T genotype and thus the persistence/non-persistence phenotype.

### LCT enhancer methylation levels are associated with genotype and phenotype

**Figure 4:**
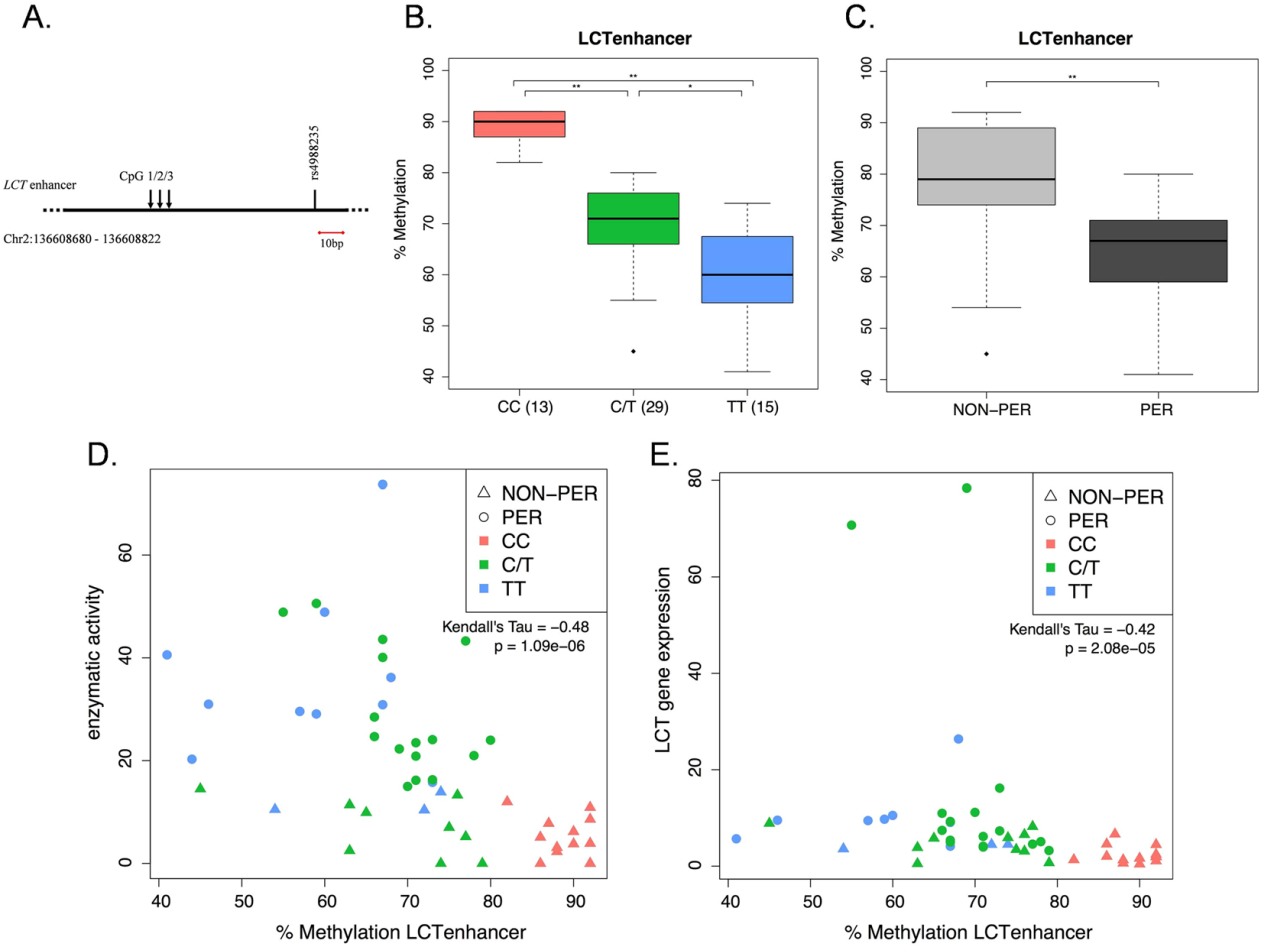
Pyrosequencing of DNA methylation at the LCT enhancer. (**A**) Schematic of *LCT* enhancer with location of the three CpGs under investigation. The position of −13910C > T (rs4988235) in relation to the CpGs and the genomic coordinates of the PCR product are also given. (**B)** DNA methylation quantification in CC vs. C/T vs. TT individuals, as well as in (**C)** Phenotypically Persistent (PER) and Non-persistent (NON-PER) individuals. Student’s T-test was calculated for inter-group comparisons (*p < 0.05; **p < 0.001). Number of individuals in each group is given in parenthesis. (**D)** Correlation between DNA methylation averaged over the three CpGs and lactase enzymatic activity (enzymatic activity data from Baffour-Awuah *et al*.^8^). (**E)** Correlation between DNA methylation averaged over the three CpGs and *LCT* gene expression (*LCT* gene expression data from Baffour-Awuah *et al*.^8^).

Given these genotype and phenotype associations with methylation at a locus in close proximity to *LCT* (cg20242066), we explored the potential impact of methylation of other regulatory elements associated with the *LCT* gene. We noted an additional association with genotype on the *MCM6* gene (cg13056269), however it lost statistical significance after SVA adjustment and we did not investigate it further (Fig. 2D). We used pyrosequencing to investigate DNA methylation at three CpGs within the *LCT* enhancer region upstream of rs4988235 (chr2:136608680-136608822, hg19; Fig. 4A) additional to the Illumina bead chip. This region corresponds to intron 13 of the *MCM6* gene, where multiple SNPs, all within a few hundred base pairs of each other, have been described not only in lactose tolerant Europeans, but also in lactase persistent pastoralist populations of Africa and the Middle East^4,28,29^. This region also overlaps with the human *MCM6* intron13-exon13 region where Labrie *et al*. previously identified inter-individual and cell-type specific variation of *LCT* mRNA levels associated with DNA modification levels^15^.

**Figure 5:**
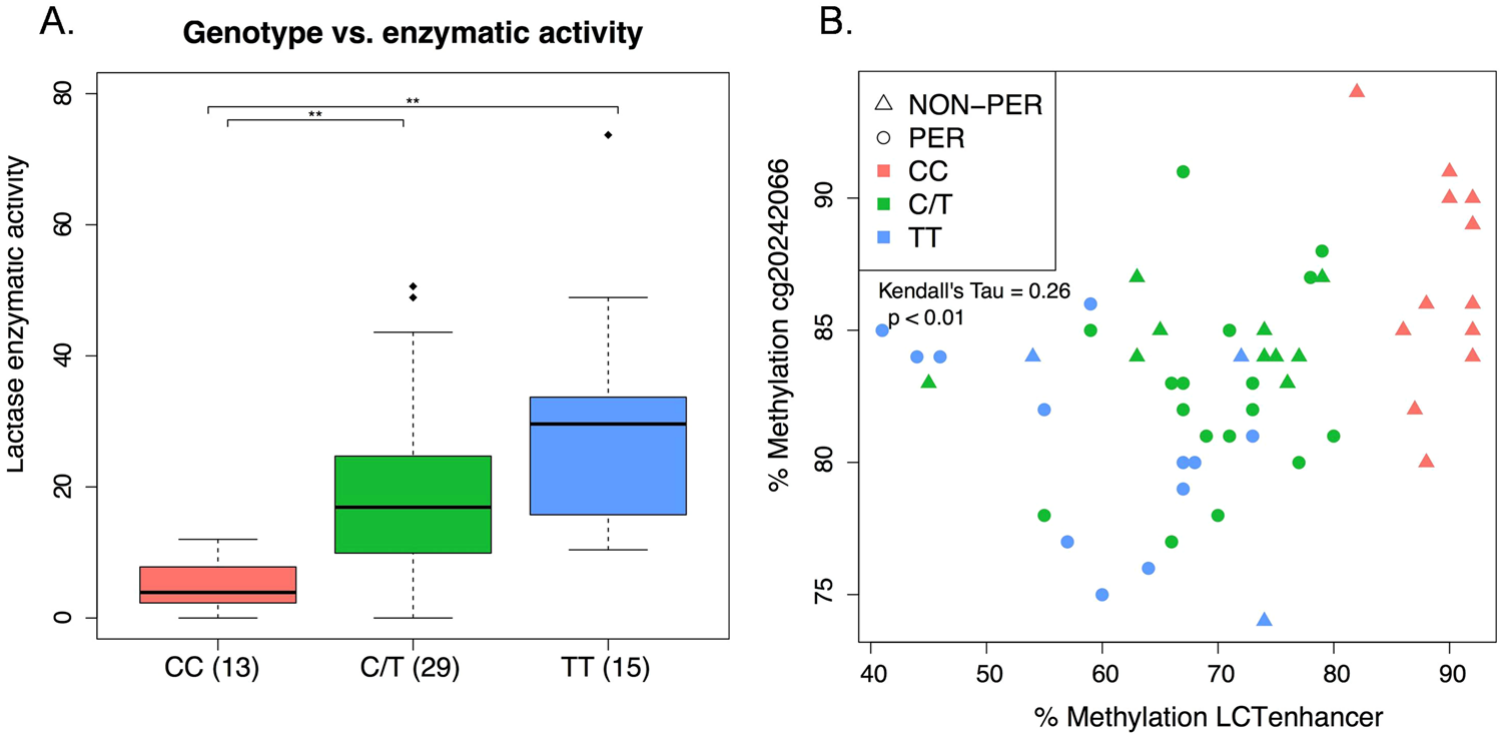
Correlation between DNA methylation at the LCT enhancer and cg20242066. (**A**) Lactase enzymatic activity according to the genotype of the individual at −13910C > T (rs4988235). Student’s T-test was calculated for inter-group comparisons (*p < 0.05; **p < 0.001). (**B)** Correlation between DNA methylation at the *LCT* promoter cg20242066 and *LCT* enhancer.

The average DNA methylation levels across these CpGs were significantly higher in CC vs. C/T vs. TT individuals and in non-persistent individuals vs. persistent (Fig. 4B,C). This effect of genotype at rs4988235 on DNA methylation variation within this region is similar to what has been previously reported for enterocytes^15^. As was the case for methylation levels on the *LCT* promoter, Kendall’s Tau correlation coefficients suggested a negative correlation between the *LCT* enhancer methylation and both the rs4988235 genotype and phenotype (correlation = −0.65; p = 9E-10 and correlation = −0.45; p = 5E-5, respectively). We also observed a negative correlation between methylation and LPH enzymatic activity (correlation = −0.48; p = 1.09E-6; Fig. 4D), and between methylation and *LCT* gene expression (correlation = −0.42; p = 2.08E-05; Fig. 4E). Of note, despite the similar correlation behavior of methylation levels at the *LCT* promoter and enhancer we only observed a weaker, albeit significant, positive correlation between the two (correlation = 0.26, p = 0.006; Fig. 5B).

### The association between DNA methylation at the lactase enhancer and lactase enzymatic activity is largely attributable to the −13910C > T genotype

Previous studies have demonstrated that the genotype at −13910C > T (rs4988235) is associated with differential lactase enzymatic activity. The CC homozygous individuals generally have low enzymatic activity, while TT homozygotes have high enzymatic activity levels. The C/T heterozygotes in this cohort mostly have intermediate values but these are heterogeneous (Fig. 5A; enzymatic activity data from Baffour-Awuah *et al*.)^8^. As DNA methylation variation is affected by genetic variation, we designed three linear models to test the association between DNA methylation at the lactase enhancer site and lactase enzymatic activity under the influence of the genotype at rs4988235. We fitted the DNA methylation obtained from pyrosequencing individually to (i) lactase enzymatic activity or (ii) the genotype and (iii) the combination of them, while adjusting all models for age and gender. Both the genotype and enzymatic activity are highly predictive for the DNA methylation (p < 0.001), with the former being more predictive than the latter having R^2^ values of 0.64 and 0.31, respectively (see Table 2 for model fit results). A full linear model combining the two predictors revealed only the rs4988235 genotype as significant (p < 0.001, adj. R^2^ = 0.65). Comparing the full to the reduced linear models showed a significant improvement only for lactase enzymatic activity (ANOVA; p < 0.001), but no improvement for genotype (ANOVA; p > 0.1). These results suggest that most of the DNA methylation variation at the *LCT* enhancer is explained by the genotype at rs4988235 alone.

**Table 2:** Linear regression analysis of pyrosequencing measurements, LPH enzymatic activity and/or the −13910C > T genotype. Asterisks indicate significance of coefficients (p < 0.1, *p < 0.05, **p < 0.01, ***p < 0.001). The table shows the predictive power of DNA methylation for all −13910C > T genotypes. The coefficients for C/T and TT are reported with respect to the CC genotype. Negative coefficients indicate lower methylation levels for the C/T and TT genotype compared to the CC genotype.

CpG location	Linear model	Enzymatic activity	C/T	TT	Sex	Age	Adjusted R^2^
LCT enhancer	Enzymatic activity	−0.40***	—	—	−5.27	0.62	0.31
	Genotype	—	−18.04***	−27.88***	−3.59	0.34	0.64
	Full	−0.11	−16.3***	−25.1***	−3.45	0.38	0.65
LCT promoter	Enzymatic activity	−0.11**	—	—	1.00	0.14	0.14
	Genotype	—	−3.54**	−6.19***	1.26	0.07	0.25
	Full	−0.04	−2.80*	−5.03**	1.32	0.09	0.26

We performed the same analysis for DNA methylation at cg20242066. In this scenario the genotype again was a significant predictor in both the partial and the full model, whereas lactase enzymatic activity was only significant in the partial model (Table 2). Testing the reduced versus the full model by ANOVA showed no improvement with respect to the genotype (ANOVA; p > 0.1), but a significant improvement for lactase enzymatic activity (ANOVA; p < 0.01). This confirms again the influence of the rs4988235 genotype on DNA methylation variation.

### Phenotype prediction with DNA methylation on the lactase enhancer/promoter and the rs4988235 genotype

**Figure 6:**
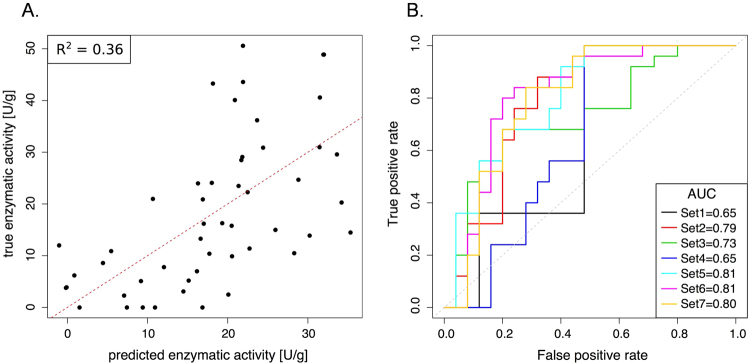
Testing the performance of LCT promoter and enhancer methylation in predicting lactase enzymatic activity levels and the bivariate phenotype lactase persistence/non-persistence. (**A**) Power of combined *LCT* promoter and enhancer methylation to predict lactase enzymatic activity levels. (**B**) Plot of Area under the curve (AUC) of the receiver-operating characteristic (ROC) calculated using genotype at −13910C > T (rs4988235), *LCT* promoter and enhancer DNA methylation, and combinations thereof (shown in detail in Table 3). Perfect prediction corresponds to AUC = 1; random prediction corresponds to AUC = 0.5. Set 1 uses rs4988235 genotype alone, while Set 6 the combination of *LCT* promoter and enhancer methylation to distinguish lactase persistors from non-persistors. In plots A. and B. one outlier with lactase enzymatic activity at 73.7 U/g was excluded from the analysis. Enzymatic activity data is from Baffour-Awuah *et al*.^8^.

Finally, we investigated the predictive ability of DNA methylation and genotype for clinical lactase persistence/non-persistence phenotype, and continuous enzymatic activity levels. Apart from the patients with low sucrase activity levels, we further excluded one outlier with high lactase enzymatic activity of 73.7 U/g protein from analysis. As potential predictors we used pyrosequencing methylation levels for the *LCT* enhancer and promoter, the −13910C > T genotype (CC, C/T and TT) as well as combinations of the three, which resulted in seven different sets of predictors (Table 3). To evaluate the prediction performance, we conducted a leave-one-out-cross-validation (LOOCV), i.e. using all patients but one for training and predicting the patient that was left out iteratively for all patients. To predict continuous enzymatic activity levels, we used a linear regression model and evaluated performance according to the cross-validated coefficient of variation R^2^ between predicted and true values. In case of lactase persistence/non-persistence, we used a logistic regression function to predict the bivariate response variable and evaluated the model performance from the receiver-operating characteristic (ROC). The best predictions for both the enzymatic activity levels (R^2^ = 0.36) and the phenotypic bivariate response (AUC = 0.81) were obtained from combining the methylation status of the *LCT* promoter and enhancer as depicted in Fig. 6A,B, respectively (Table 3). Quite surprisingly, the methylation levels of the *LCT* promoter and enhancer outperform the predictive power of the rs4988235 genotype (R^2^ = 0.24, AUC = 0.65) or genotype/methylation combination (R^2^ = 0.29, AUC = 0.80) for the continuous or bivariate readout. Thus, methylation must play an important and direct role in determining the *LCT* gene expression and lactase activity, and can predict the persistence/non-persistence phenotype, especially for the heterogeneous mixed C/T genotype.

**Table 3:** Prediction performances for enzymatic activity levels and lactase persistence/non-persistence phenotype using different sets of predictors: *LCT* enhancer and promoter are averaged methylation levels in their respective location based on DNA methylation measurements from pyrosequencing. The −13910C > T genotype consists of categories CC, C/T and TT.

Set	Predictors	R^2^ true vs. predicted enzymatic activity levels	AUC of lactase persistence/non-persistence prediction
Set1	Genotype	0.24	0.65
Set2	LCTenhancer (Methylation)	0.30	0.79
Set3	LCTpromoter (Methylation)	0.15	0.73
Set4	Genotype + LCTenhancer	0.26	0.65
Set5	Genotype + LCTpromoter	0.24	0.81
Set6	LCTenhancer + LCTpromoter	0.36	0.81
Set7	Genotype + LCTenhancer + LCTpromoter	0.29	0.80

## Discussion

In this study, we have investigated molecular mechanisms at the transcriptional and epigenome level that might control *LCT* expression, and maintain decreased levels of jejunal lactase throughout adulthood in the majority of the human population. Human *LCT* is activated in the fetal intestine late in embryonic development and reaches peak expression at 40 weeks gestation^30^. Physiological down-regulation of lactase expression in humans occurs post-weaning in the absence of intestinal injury or disease. Furthermore, lactase phlorizin hydrolase is not affected by dietary changes and lactose intake. LPH is not reduced by the absence of lactose in the diet^31^; neither does lactose feeding induce lactase enzymatic activity and gene expression^32^.

It is accepted that lactase gene expression is primarily regulated at the transcriptional level^33–35^, and in non-human mammals Cdx2, Gata4/6 and Hnf1α TFs collectively activate this gene. However, we did not observe any of these to be differentially expressed in intestinal biopsies from lactase persistent and non-persistent individuals. The −13910C > T variant (rs4988235) has previously been shown to be associated with lactase persistence in people of European ancestry, the T-allele being responsible for functional persistence of the lactase promoter^7,18^. Notably, when we stratified our cohort according to genotype at rs4988235, CDX2 expression was lower in TT homozygotes. In addition, we found an increase in GATA4 expression to be associated with LPH enzymatic activity, although we did not observe an association between GATA4 expression and lactase gene expression levels. As we did not observe differential TF regulation, post-translational processing and complex glycosylation of the protein that *LCT* codes for should influence lactase abundance and enzymatic activity^36–38^. The association between GATA4 expression level and LPH enzymatic activity, therefore, might be rather through regulation of genes important for LPH post-translational modification and activation.

Using an unbiased epigenome-wide approach verified by targeted pyrosequencing, we have identified position cg20242066 (chr2: 136595261; hg19) as differentially methylated in the jejunum of lactase persistent and lactase non-persistent children and adolescents. This CpG is located in the region essential for spatio-temporal regulation of human *LCT* expression, which has been previously demonstrated by introducing a construct containing the sequence 3.3kbs upstream of the TSS in transgenic mice^39^. Linear regression analysis showed that DNA methylation at both the *LCT* promoter and enhancer is largely impacted by genotype at −13910C > T, with CC homozygotes having the highest, and TT homozygous individuals the lowest methylation levels. Most importantly, however, cross-validation analysis revealed that methylation at the *LCT* promoter and enhancer was highly predictive of lactase enzymatic activity, and the persistence/non-persistence phenotype. The predictive power outperformed the hitherto existing genotype at rs4988235, which fails prediction for the C/T genotype. Hence, promoter and enhancer methylation might be a valuable biomarker for phenotype prediction. This reflects the existing crosstalk between DNA methylation and genetic variation, which has been reported previously in population studies^40–42^. Indeed, DNA methylation patterns associated with specific genotypes (i.e. meQTLs) have consistently been shown to influence gene expression and disease phenotypes^43–45^. Interestingly, a recent study by Banovich *et al*. in lymphoblastoid cell lines demonstrated that SNPs, which affect transcription factor binding affinities are associated with DNA methylation at CpGs in proximity^46^. In that study it was suggested that changes in TF binding are an early regulatory step leading to subsequent molecular changes, including epigenetic. Differential binding of POU2F1, preferentially to the -13910*T-allele, which is associated with lactase persistence^18^ might be acting in much the same way. Within the highly conserved lactase TATA-box promoter a differentially methylated position, such as cg20242066, might function to preferentially recruit regulatory proteins. However, no major intestinal transcription factor consensus sequences appear to be close to this CpG.

Prior to SVA adjustment our list of top 20 DMPs associated with genotype revealed cg13056269 (chr2:136633607; hg19), a CpG site located in intron 1 of the upstream *MCM6* gene. In the future, it would be interesting to investigate whether intron 1 of *MCM6* might also affect *LCT* gene expression, as does intron 13 of the same gene where −13910C > T (rs4988235) and the *LCT* enhancer are located.

It is important to mention that cg20242066 was not identified as differentially methylated by Labrie *et al*. (see their Supplementary Table 1)^15^. The authors of that study used a library-free version of BSPP (bisulfite padlock-probe sequencing)^47^, which allows interrogation of DNA methylation at single nucleotide resolution, but is limited by the position and efficiency of the probes that can be designed. The efficiency of the probes depends on the underlying target sequence, including its CG content and length^48^. A search in the list of human probes used in Labrie *et al*. did not reveal the CpG that we report on here.

In summary, we have investigated the contribution of epigenetics to the lactase persistence/non-persistence phenotypes, including lactase gene expression and enzymatic activity. We have identified putative lactase meQTLs, which are differentially methylated between lactase persistent and lactase non-persistent individuals. In genetically homogenous populations, it appears that the T-allele at −13910C > T might be dominant leading to a trimodal distribution in lactase enzymatic activities and the complete correlation of lactase persistence with the presence of the variant allele. However, in genetically heterogeneous populations, such as the one discussed here, we observe that this likely is not the case. Methylation at the *LCT* enhancer and the *LCT* promoter are both affected by the genotype at rs4988235, and appear to be continuously associated with lactase phenotypes. Combining them outperforms the previously known genotype when it comes to predicting the lactase persistence vs. lactase non-persistence phenotype and the lactase enzymatic activity levels. Despite the fact that we have attempted to remove any unwanted sources of inter-sample variation, we cannot entirely exclude the possibility that such variation exists and has remained unaccounted for even after surrogate variable analysis. Our observation of a closer correlation of DNA methylation with phenotype (as assessed by enzyme activity), than correlation of genotype with enzyme activity, could theoretically be a reflection of that variation, irrespective of the fact that the biopsies were deemed normal by histology. However, as Labrie *et al*. have previously described^15^, the regions surrounding the *LCT* gene where DNA methylation variation associated with lactase persistence/non-persistence was detected, overlap with DNaseI hypersensitivity sites, which carry histone marks of active enhancers. Hence, DNA methylation levels at the *LCT* enhancer and promoter are reflective of additional layers of epigenetic regulation, which might explain their improved performance in prediction of lactase phenotypes.

## Materials and Methods

### Study population

Mucosal pinch-biopsy samples were obtained from the third portion of the duodenum in patients (ages 8 to 23 years) undergoing diagnostic esophago-gastroduodenoscopy at Boston Children’s Hospital between 2005 and 2007 as described in detail by Baffour-Awuah *et al.*^8^. Indications for endoscopy included dysphagia, chronic abdominal pain, symptoms of gastroesophageal reflux and esophagitis. Patients with known small intestinal disorders such as celiac disease, diarrhea or other pathological conditions, such as gastrointestinal bleeding, inflammatory bowel disease, or immunodeficiency were excluded. Signed informed consent/assent was obtained from each patient and his/her parent or legal guardian who agreed to participate in the initial, and any follow-up, studies. The samples were snap-frozen and stored at −80 °C. Two biopsy samples were submitted for histological analysis; one biopsy was saved for total protein and disaccharidase assays, and the other samples were available for RNA and DNA analysis, as reported (Baffour-Awuah *et al*.), and epigenetic assays as described below. Permission to use a subset of these samples (aged 8 to 19) for the study of epigenetic regulation of lactase was granted following review by Partners Human Research IRB Committee at Brigham and Women’s Hospital in Boston and the Committee on Clinical Investigation at Boston Children’s Hospital in Massachusetts, USA. All experiments were performed in accordance with relevant guidelines and regulations, including the removal of any identifying information.

Examination of biopsies by two experienced GI pathologists revealed normal villus height and crypt depth, and normal cellular distribution, with an absence of intraepithelial lymphocytes or abnormal inflammatory cells in the *lamina propria*. Standards for morphological assessment of intestinal biopsies have been previously published^49^.

Subjective reporting of lactose intolerance was obtained by history from each subject. Only 3 patients included in this study were lactose intolerant by report. Disaccharidase analysis were assayed in a CLIA certified laboratory as originally reported^8^ using the method of Dahlqvist^50^. Quality control for disaccharidase assays at the Gastrointestinal Laboratory of the Buffalo Children’s Hospital (Kaleida Health; Buffalo, NY) is achieved using standard procedures. At least annually, masked, randomly selected, frozen samples are submitted to a reference laboratory where they are assayed in triplicate by the Dahlqvist method. Results are generally identical to the value originally obtained for that sample in the submitting laboratory (within only a 0.5% variation).

Ranges of disaccharidase activity, Sucrase/Lactase ratios and definitions of lactase persistence and non-persistence were similar to those in previously published studies^1,51–53^. Therefore, cut-point values of lactase < 15 U/g protein and S/L ratio > 2 was considered lactase non-persistence, while lactase activity > 15 U/g and S/L < 2 was considered lactase persistence. As published previously, lactase activity did not change with age in this sample population^8^.

### DNA isolation

Genomic DNA was isolated using DNeasy Blood and Tissue Kit by QIAGEN (#69506). Briefly, a small tissue biopsy was incubated at 56 °C in 180 uL Lysis Buffer supplemented with 20 uL Proteinase K solution for 2 hrs, or until the tissue was completely dissolved. As per the manufacturer’s protocol, 200 uL AL Buffer was pre-mixed with 100% ethanol in 1:1 ratio and added to each sample. The samples were then loaded onto the supplied columns and washed. Each sample was eluted twice in a total volume of 150 uL Elution Buffer. DNA quantity and quality was measured on a NanoDrop 1000 Spectrophotometer.

### Genome-wide DNA methylation analysis

Genome-wide profiling of DNA methylation was performed using the Infinium Human Methylation 450K BeadChip at the University of Southern California Norris Comprehensive Cancer Center Molecular Genomics Core Facility. This methylation microarray surveys the DNA methylation status of 482,421 CpGs in the human genome. It interrogates approximately 99% of RefSeq genes including promoter, 5′ and 3′ gene body regions, as well as CpG sites located outside of gene coding regions. The probes are located within 80% of core promoters, 94% of distal promoters and 97% of all gene bodies. Coverage is provided for 94% of all CpG islands and 93% of all CpG island shores in the human genome.

Briefly, 1 µg genomic DNA for each sample was treated with sodium bisulfite, recovered using the Zymo EZ DNA Methylation Kit (Zymo Research, Irvine, CA) according to the manufacturer’s specifications and eluted in 18 uL volume. An aliquot (3 uL) was removed for MethyLight-based quality control testing of bisulfite conversion completeness and the amount of bisulfite converted DNA available for the Infinium Methylation assay. All samples that passed the QC tests entered into the Infinium DNA methylation assay data production pipeline. Data processing was performed at the USC High Performance Computing Center (HPCC) using dedicated, Linux-based, high performance computational cluster and enterprise storage. After the chemistry steps, Bead Arrays were scanned and the raw signal intensities extracted from the *.IDAT files using the Bioconductor *minfi* (1.12.0) package. The intensities were corrected for background fluorescence followed by dye-bias normalization. The beta values were calculated as M/(M + U + 100) as recommended by Illumina, in which M and U refer to the (pre-processed) mean methylated and unmethylated probe signal intensities, respectively. M-values were calculated as logit transformed beta values. Measurements in which the fluorescent intensity was not statistically significantly above background signal in at least 10% of the samples (detection p value > 0.01) or with bead counts ≤ 3 were removed from the data set. In addition, probes that overlap with known SNPs as well as repetitive elements were masked prior to data analyses: specifically, all HM450 probes that overlapped with common SNPs with a minor allele frequency of greater than 1% (UCSC criteria) at the targeted CpG site, as well as probes with common SNPs (MAF >1%) within 10 bp of the targeted CpG site. HM450 probes that were within 15 bases of the CpG lying entirely within a repeat region were also masked prior to data analyses.

### Statistical analysis of the 450K methylation array

Prior to statistical analysis, sample outliers were removed based on the median methylated and unmethylated intensities (Fig. 2B), and CpG sites located on the X- and Y-chromosomes were masked to reduce gender effects. For phenotypic analysis, samples with sucrase activity levels <20 U/g were further excluded. Due to variation of technical replicates, we performed batch correction on the 450K lanes using ComBat^27^. Statistical analysis was performed on M-values as proposed in Du *et al*.^54^. To identify differentially methylated positions between groups or continuous variables, F-tests were calculated adjusting for age and gender using the *limma* pipeline^55^. In addition, in order to account for sources of unmodeled DNA methylation heterogeneity, we used surrogate variable analysis (SVA) as originally described by Leek *et al*.^56,57^. Heatmaps show z-score transformed M-values and are ordered based on Euclidean distance and average clustering. Associations among methylation and covariates were measured using Kendall’s Tau correlation.

### Multiple linear regression analysis

Linear regression models were evaluated using the lm function from the stats package, where two-tailed p-values for covariates were obtained from the t-statistic. The adjusted R^2^ was calculated according to Wherry formula and linear models were compared by ANOVA.

### Prediction performance of enzymatic activity levels and bivariate phenotype

Prediction was evaluated using LOOCV with a linear regression model for continuous enzymatic activity levels and a logistic regression model for the bivariate phenotype (persistence/non-persistence). To rank performances we used the coefficient of variation R^2^ between predicted and true enzymatic activity levels as well as the AUC for the bivariate response. ROC curves and AUC were calculated using the ROCR package^58^. All analyses were performed using the statistical program language R^59^.

### Bisulfite conversion of DNA for Pyrosequencing

Bisulfite conversion of DNA was carried out using the EZ-DNA Methylation Gold Kit by Zymo Research (#D5006). Briefly, 500–1000 ng of genomic DNA in 20 uL was incubated with 130uL conversion reagent at 98 °C/10 min, followed by incubation at 64 °C for 2.5 hrs. The samples were then passed through columns, desulphonated for 20 min, washed and eluted with 15 uL elution buffer. This bisulfite converted DNA was stored at −20 °C and used for downstream PCR reactions.

### Pyrosequencing

Genotyping of rs4988235 (−13910C > T), analysis of DNA methylation at the *LCT* enhancer and verification of the 450K results were performed by pyrosequencing on a PyroMark Q24 instrument (QIAGEN). Primers for all pyrosequencing assays were designed using the PyroMark Assay Design software 2.0 and purchased from Integrated DNA Technologies (IDT). All biotinylated primers were HPLC purified. Detailed information on the pyrosequencing assays and procedures is provided in Supplementary Table 2. Pyrosequencing was carried out according to the manufacturer’s instructions (QIAGEN). Briefly, biotin labeled PCR products were incubated with Streptavidin Sepharose High Performance Beads (#17–5113–01; GE Healthcare Life Sciences) in the presence of PyroMark Binding Buffer (#979006) for 5–10 min at room temperature and constant agitation. The beads were then captured by the vacuum tool of the PyroMark workstation and washed consecutively in 70% ethanol, PyroMark Denaturation Solution (#979007) and 1 × PyroMark Wash Buffer (#979008). The biotin-tagged single-strand DNA was released onto the wells of PyroMark Q24 plates (#979201, QIAGEN) containing the respective sequencing primer diluted to a final concentration of 300 mM in PyroMark Annealing Buffer (#979009). The plate was then incubated at 80 °C/2 min and allowed to cool to room temperature before being placed into the PyroMark Q24 instrument. Pyrosequencing was performed using nucleotides, enzyme and substrate solutions provided in PyroMark Gold Q24 Reagents (#970802, QIAGEN) which were loaded in a PyroMark Q24 Cartridge (#979201, QIAGEN).

### Reverse-Transcription quantitative PCR

For quantitative RT-qPCR analysis we excluded samples with sucrase-isomaltase (SI) levels < 25 U/g protein, according to previously established guidelines^25^. Exon-intron boundary-spanning primers were designed using the Primer3 v.4.0 software and purchased from IDT. cDNA was synthesized from 1000 ng of total RNA using the High Capacity cDNA Reverse Transcription Kit (Life Technologies) and the provided random primers. Negative controls containing no reverse transcriptase were included. Each primer pair was optimized for robust and target-specific amplification. Following optimization, qPCR reactions were carried out in duplicate on 96-well plates using a Mastercycler ep Realplex instrument (Eppendorf). A detailed list of RT-qPCR primers and conditions can be found in Supplementary Table 3. Raw fluorescence values were exported from the Realplex 2.0 software following each run. These were used to establish baseline fluorescence and to analyze the amplification curves with the LinRegPCR software^60,61^. Relative gene expression was quantified by normalization to two reference genes, GAPDH and ACTIN, using the model described in Pfaffl, *Nucleic Acids Research*^62^. We excluded samples that showed signs of inhibition of RT-qPCR during amplification of the reference genes. A pool of total RNA from adolescent male jejunum was used as a calibrator (H.jej.). The relative expression level of each target gene was calculated by taking into account the efficiency (E) of amplification, and the quantification cycle (Cq) deviation of the sample versus the calibrator, in comparison to the reference genes using the formula:

$${\rm{Relative}}\,{\rm{expression}}=\frac{{\rm{TargetGene}}\,{{\rm{E}}}^{(Cq(H.jej.)-Cq(sample))}}{{\rm{AvgRefGene}}\,{{\rm{E}}}^{(GeoMeanRefGeneCq(H.jej.)-GeoMeanRefGeneCq(sample))}}$$

### Statistical analysis of RT-qPCR

Gene expression levels of all of the investigated genes were approximately normally distributed in our cohort (Supplementary Figure S1b). Co-expressions among the TFs were measured using Spearmans rank correlation. Correlations between TF expression and continuous phenotypes were assessed using Pearson correlations, whereas associations with dichotomous or polytomous variables were analyzed using a t-test or one-way ANOVA, respectively. Linear models were used to assess the impact of adjustment for gender and age on the associations between TF expression, and both enzymatic activity and *LCT* expression. Statistical analysis was performed using R version 3.2.1 and data was visualized using ggplot2.

## Electronic supplementary material

Supplementary Information
