# Supplementary material for: Differences in DNA Methylation and Functional Expression in Lactase Persistent and Non-persistent Individuals
Source: Sci Rep. 2018 Apr 4;8:5649. doi: 10.1038/s41598-018-23957-4 (PMC5884863; doi:10.1038/s41598-018-23957-4)
Supplement: Supplementary file 1 — Supplementary Information [file 41598_2018_23957_MOESM1_ESM.pdf]

Differences in DNA Methylation and Functional Expression in Lactase Persistent and Non-persistent Individuals

Milena N. Leseva [1]

Richard J. Grand [2]

Hagen Klett [3,4,5]

Melanie Boerries [3,4,5]

Hauke Busch [3,8]

Alexandra M. Binder [6]

Karin B. Michels [1,6,7]

[1]*Institute for Prevention and Cancer Epidemiology, Faculty of Medicine and Medical Center, University of Freiburg, Germany*

[2]*Division of Gastroenterology and Nutrition, Boston Children's Hospital, Harvard Medical School, Boston Massachusetts, USA*

[3]*Institute of Molecular Medicine and Cell Research, University of Freiburg, Germany.*

[4]*German Cancer Consortium (DKTK), Freiburg, Germany.*

[5]*German Cancer Research Center (DKFZ), Heidelberg, Germany*

[6]*Obstetrics and Gynecology Epidemiology Center, Department of Obstetrics, Gynecology and Reproductive Biology, Brigham and Women's Hospital, Harvard Medical School, Boston Massachusetts, USA*

[7]*Department of Epidemiology, Harvard School of Public Health, Boston, Massachusetts, USA*

[8]*Luebeck Institute of Experimental Dermatology – Institute for Cardiogenetics, Luebeck, Germany*

Correspondence should be addressed to K.B.M. (email: kmichels@hsph.harvard.edu)

Supplementary Figure Legends

Figure S1. Characteristics of samples included in RT-qPCR analysis. a. Disaccharidase activity levels in lactase persistent (>15U/g) and lactase non-persistent (<15U/g) individuals (data from Baffour-Awuah *et al.*, 2015). Log_2 of S/L ratios are given for lactase persistent and non-persistent individuals according to the above definition. **b.** Distribution of CDX2, POU2F1, GATA4, GATA6, and HNF1α expression levels in our cohort.

Figure S2. Principal component analysis prior to batch correction of 450K DNA methylation array.

Figure S3. Genome-wide DNA methylation analysis of lactase persistent and non-persistent individuals after adjusting for sucrase enzymatic activity levels. Colorspace corresponds to methylation levels as z-score transformed M-values (yellow = hypo-, blue = hypermethylation). **a.** Top 20 DMPs identified following ordinal regression of genotype (CC=0, C/T=1, TT=2); **b.** Top 20 DMPs identified from ordinal regression analysis using quartiles of enzymatic activity levels (q1=[0, 6.8], q2=[6.8, 14.8], q3=[14.8, 28.6], q4=[28.6, 73.7]). Both analyses were adjusted for sex, age, inter-sample differences in sucrase enzymatic activity levels and estimated surrogate variables.

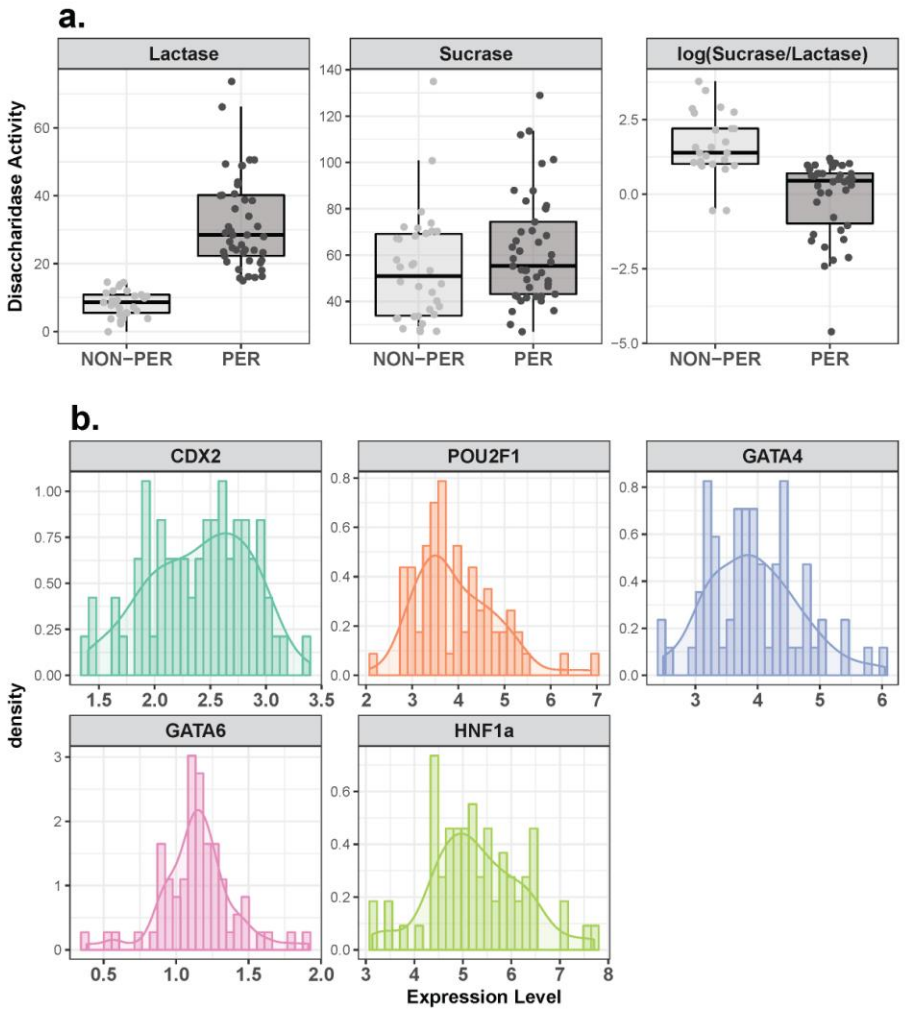

Figure S1.Characteristics of samples included in RT-qPCR analysis.

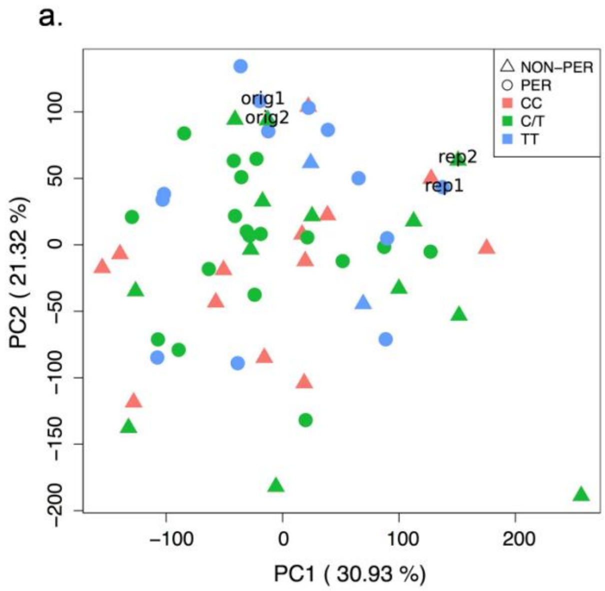

Figure S2. Principal component analysis prior to batch correction of 450K DNA methylation array

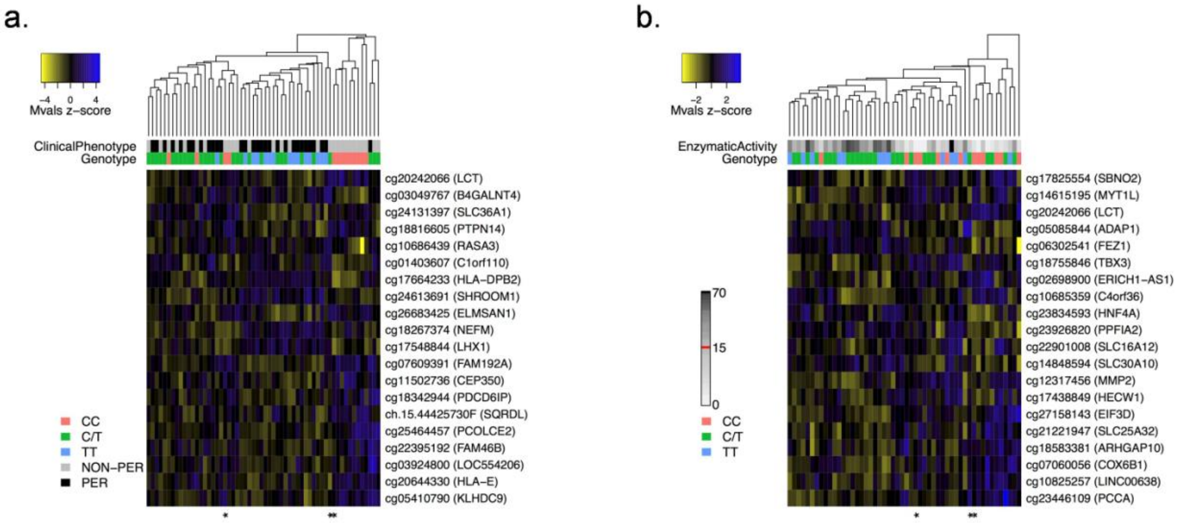

Figure S3. Genome-wide DNA methylation analysis of lactase persistent and non-persistent individuals after adjusting for sucrase enzymatic activity levels.

Supplementary Table1 Ancestry information on each subject included in the 450K

DeID	Mother	Father
1[b]	Scotland	Unknown
2	Italy	N. Europe/N. America
3	Italy	Scotland/England/N. Europe
4	England	France
5[b]	Unknown	Unknown
6[b]	Ukraine	Unknown
7	Sub-Saharan Africa	Sub-Saharan Africa
8	Lithuania	Canada
9	Northern Europe	Northern Europe
10	Northern Europe	Northern Europe
11[b]	Unknown [Caucasian]	Unknown
12[b]	England/Ireland	Unknown
13	Germany/England	N. Europe (Scotland/England)
14	Netherlands/Germany	England/Ireland
15	Scotland/Canada	Canada
16	Lithuania/Russia	Russia
17	Ireland/Native America	Scotland
18	Italy/England/Germany	Ireland/Greece/Denmark/England
19	Spain/Puerto Rico	Puerto Rico
20	Spain	Germany
21[a]	Ireland	Ireland
22	French Canadian/Italy	England/Wales
23	Puerto Rico	Germany/Norway
24	Ireland	Wales/ French Canadian
25	Poland	Ireland
26[b]	Unknown	Unknown
27	Europe	Germany
28	Peru	Ireland
29	Ireland	Ireland/Portugal
30	Germany	Ireland
31	China	China
32	Eastern Europe	Eastern Europe
33	Italy/Jewish	Cape Verde
34	N. America/Canada	Puerto Rico
35	Ireland	Lebanon
36	Ireland	England/Ireland/Lithuania
37[b]	Northern Europe	Unknown
38[a]	Sweden	Ireland
39	Syria	Syria

40	Germany	Poland/England
41	Portugal/Ireland	Germany/North America
42	Ireland/England/Scotland	England/Ireland/Scotland
43[b]	Unknown	Unknown
44[a]	Sweden/England	Italy/Northern Europe
45	Portugal	Latvia
46	Ireland/England	Portugal
47	Puerto Rico	Puerto Rico
48	Poland	Greece
49	Germany	Ireland/England
50[b]	Unknown	Unknown
51	England	England/Ireland
52	Russia	Germany
53	Portugal/England/Ireland	Greece
54	Ireland	French Canadian
55	Italy/Albania/England/Ireland	Ireland/Canada
56	Germany	Italy
57	N. America/Sweden/Germany	France
58	Ireland	Italy/France
59	Italy/Ireland	Lithuania/Germany/England
60	Canada/Northern Europe	Canada/Northern Europe

[a] lactase non-persistent -13910*TT individual

[b] missing ancestry information

Supplementary Table 2 Pyrosequencing information

**General information**	
Pyrosequencer	PyroMark Q24 (Qiagen)
Individual binding reaction	PCR product: 20-25uL<br>Streptavidin Sepharose High Performance Beads (GE Healthcare Life Sciences): 2uL<br>Binding Buffer (Qiagen): 40uL<br>Nuclease-free water (IDT): 18uL
Individual sequencing reaction	Total volume: 25uL<br>Sequencing primer: 300nM final concentration in Annealing Buffer (Qiagen)
**Genotyping rs4988235 (-13910C>T)**	
Primer sequences	For(biotin):TGCGCTGGCAATACAGATA<br>Rev: GAATGCAGGGCTCAAAGAAC<br>Seq: GCAACCTAAGGAGGAGA
PCR reaction	Reaction volume: 25uL<br>Genomic DNA (25ng/uL): 2uL<br>Primers: 250nM final concentration each<br>Polymerase, nucleotides and buffer included in 2X MegaMix-Gold HotStart PCR Master mix (2MMG-50; Gel Company)
Thermo-cycler and conditions	Applied Biosystems® 2720 Thermal Cycler (Life Technologies)<br>Initial denaturation: 95°C/5min<br>50 cycles: 95°C/30sec<br>Ta=56.5^{O}C/30sec<br>72°C/45sec<br>Final extension: 72°C/7min<br>Hold: 4°C
PCR product length	113bp
Sequence to analyze	5'GTTCCTTTGAGGCCAGGG**G/A**CTACATTATCTTATCTGT ATTGCCAGCGCAGAGGCC3'<br>*rs4988235 in bold
**LCT enhancer (chr2: 136608680-136608822; genome build hg19)**	

Primer sequences	For: AAGAGTTTGGTAAGTATTTGAGTG<br>Rev(biotin): AAACCTACTAATACATTATAAAATCTAAAT<br>Seq: GGTAAGTATTTGAGTGTAG
PCR reaction	Reaction volume: 25uL<br>Bisulfite converted DNA: 2uL<br>Primers: 250nM final concentration each<br>Polymerase, nucleotides and buffer included in 2X MegaMix-Gold HotStart PCR Master mix (2MMG-50; Gel Company)
Thermo-cycler and conditions	Applied Biosystems® 2720 Thermal Cycler (Life Technologies)<br>Initial denaturation: 95°C/5min<br>50 cycles: 95°C/30sec<br>Ta=54°C/30sec<br>72°C/45sec<br>Final extension: 72°C/7min<br>Hold: 4°C
PCR product length	144bp
Sequence to analyze	5'TTGTTAGA**YG**GAGA**YG**ATTA**YG**TTATAGTTTATAGAGTGTATAAAGAYGTAAGTTATTATTTAATATTTTTTAT3'<br>*CpGs analyzed in bold
**LCT promoter cg20242066 (chr2: 136595152-136595345; genome build hg19)**	
Primer sequences	For: AGTTTGGGTAATAGAATGAGATTTTA<br>Rev(biotin): ACTCCCACCTAAACAACAAAATA<br>Seq: ATTTTTTATATAAGTTAAGTAGAGG
PCR reaction	Reaction volume: 25uL<br>Bisulfite converted DNA: 1uL<br>Primers: 250nM final concentration each<br>Polymerase, nucleotides and buffer included in 2X MegaMix-Gold HotStart PCR Master mix (2MMG-50; Gel Company)
Thermo-cycler and conditions	Applied Biosystems® 2720 Thermal Cycler (Life Technologies)<br>Initial denaturation: 95°C/5min<br>50 cycles: 95°C/30sec<br>Ta=59.5^{O}C/30sec<br>72°C/45sec<br>Final extension: 72°C/7min<br>Hold: 4°C
PCR product length	193bp
Sequence to analyze	5'AATT**YG**GAAAATGTTTGAAGAAAAYGATTATTAATTTTTTTTGTTTTTTTTGTTTTTT3'<br>*cg20242066 in bold

Supplementary Table 3 Quantitative RT-PCR information

**Samples**	
Type of samples	RNA isolated from mucosal pinch-biopsies obtained from the distal end of the third portion of the duodenum.
**Nucleic acid extraction was performed by Baffour-Awuah et al.(*8*)**	
Method of isolation<br>DNase treatment<br>Storage of total RNA<br>Quantification	RNeasy mini Kit (Qiagen)<br>DNA*free* Kit (Ambion)<br>-80°C<br>NanoDrop 1000 Spectrophotometer
**Reverse transcription**	
Procedure	High capacity cDNA Reverse Transcription Kit (Life Technologies)
Individual reaction setup	Reaction volume: 20uL<br>RNA: 1000ng<br>10X RT Buffer: 2uL<br>dNTPs (100mM): 1uL<br>10X RT Random Primers: 2uL<br>MultiScribe (50U/uL) Reverse Transcriptase: 1uL
Thermo-cycler and conditions	Applied Biosystems® 2720 Thermal Cycler (Life Technologies)<br>25°C/ 10min<br>37°C/ 2hrs<br>85°C/ 5min<br>Hold/ 4°C
Storage of cDNA	-20°C
**RT-qPCR primer sequences**	
CDX2 (product length: 124bp)	F: GCTGGAGCTGGAGAAGGAGTT<br>R: CTCCTTTGCTCTGCGGTTCT
GATA4 (product length: 125bp)	F: CAACTCCAGCAACGCCACC<br>R: ACATCGCACTGACTGAGAACGTC
GATA6 (product length:123bp)	F: CCTCAGCCGGCCCCTCATCAA<br>R: GGTTCACCCTCGGCGTTTCTGC
HNF1a (product length: 147bp)	F: GAGAGGCAGAAGAACCCTAGC<br>R: CCAGTTGTAGACACGCACCTC
POU2F1 (product length: 187bp)	F: GTGCTCTCAGCCCAGCTCTAA<br>R: GTGAGCAGAGAGAGGTTCTGAGG
b-ACTIN (product length: 149bp)	F: TTGCCGACAGGATGACAGAA<br>R: GCTGATCCACATCTGCTGGAA
GAPDH (product length: 115bp)	F: CATCTTCTTTTGCGTCGCCA<br>R: TTAAAAGCAGCCCTGGTGACC

**RT-qPCR protocol**	
Fluorescence detection	Fast SYBR Green 2X Master Mix (Life Technologies)
Individual reaction set up	Reaction volume: 20uL<br>cDNA (1ng/uL): 5uL<br>Primer mix (250nM each primer): 5uL<br>SYBR Green 2X: 10uL
Thermo-cycler and conditions	Mastercycler ep Realplex (Eppendorf)<br>Hold: 95°C/5min<br>40 cycles: 95°C/30sec<br>60°C/ 1min (plate read)<br>Melting curve: 60°C to 95°C (0.5^{O}C increments)
**RT-qPCR Efficiency (E)**	
CDX2	1.88
GATA4	1.82
GATA6	1.80
HNF1α	1.88
POU2F1	1.84
Beta-ACTIN	1.86
GAPDH	1.91
